# Factors associated with the utilization and costs of health and social services in frail elderly patients

**DOI:** 10.1186/1472-6963-12-204

**Published:** 2012-07-19

**Authors:** Sari Kehusmaa, Ilona Autti-Rämö, Hans Helenius, Katariina Hinkka, Maria Valaste, Pekka Rissanen

**Affiliations:** 1 Research Department, Social Insurance Institution of Finland, Helsinki, Finland; 2 Department of Biostatistics, University of Turku, Turku, Finland; 3 Tampere School of Public Health, University of Tampere, Tampere, Finland

## Abstract

**Background:**

Universal access is one of the major aims in public health and social care. Services should be provided on the basis of individual needs. However, municipal autonomy and the fragmentation of services may jeopardize universal access and lead to variation between municipalities in the delivery of services. This paper aims to identify patient-level characteristics and municipality-level service patterns that may have an influence on the use and costs of health and social services of frail elderly patients.

**Methods:**

Hierarchical analysis was applied to estimate the effects of patient and municipality-level variables on services utilization.

**Results:**

The variation in the use of health care services was entirely due to patient-related variables, whereas in the social services, 9% of the variation was explained by the municipality-level and 91% by the patient-level characteristics. Health-related quality of life explained a major part of variation in the costs of health care services. Those who had reported improvement in their health status during the preceding year were more frequent users of social care services. Low informal support, poor functional status and poor instrumental activities of daily living, living at a residential home, and living alone were associated with higher social services expenditure.

**Conclusions:**

The results of this study showed municipality-level variation in the utilization of social services, whereas health care services provided for frail elderly people seem to be highly equitable across municipalities.

Another important finding was that the utilization of social and health services were connected. Those who reported improvement in their health status during the preceding year were more frequently also using social services. This result suggests that if municipalities continue to limit the provision of support services only for those who are in the highest need, this saving in the social sector may, in the long run, result in increased costs of health care.

## Background

The population in European societies grows older and the importance of health and social services will increase. The elderly are disproportionate users of the health and social care system and this provides a major challenge to the planning of services for older people. Traditionally, the health care and social service sectors have operated separately. The growing demand for integrated services and the rising costs are leading to a more intensive collaboration between the health care and social service providers [1].

In Finland, social and health services are largely financed with public funds, and the principal goal is that services are equitably accessible to everyone [2]. The stated national targets for services for over 75 years old people are: 92% are living at home independently or using appropriate health and welfare services, 14% are receiving regular home care, 5–6% are receiving informal care-support, and 8–9% are living in sheltered housing with 24-hour assistance or in long-term care in health centre hospitals [3].

When developing elderly care policies we need to know why people are using health and social care, and which factors are related to equitable access to care. Andersen’s behavioural model of health service use [4,5] provides a useful theoretical framework for assessing the multiple dimensions of access to care. The model contains three sets of predictive factors: predisposing, enabling and need factors for explaining health services utilization. The model has been found useful in gerontological research as it can be adapted according to the research question [6][7][8][9]. In our study we applied behavioural model to a sample of frail elderly persons to assess their use of services.

Previous studies have shown that age, functional ability, health status, chronic illnesses, and socio-economic factors have an influence on service utilization [6,10-18][19]. The variation in service utilization may be considered appropriate when it occurs due to need-related factors like functional ability or health status. When variation is found in the use of services by regions after controlling for patient characteristics, inequities might exist in the distribution of care services. This may reflect variation in the supply of services and have a detrimental effect on the health outcomes [20]. According to Andersen, unequal access occurs when social structure, health beliefs and enabling resources determine who will receive care [5].

Access to care is a multidimensional concept, and the utilization of services describes the use as “realized access” [4,20]. Even when access is thought as being equal, individuals may make different choices in relation to the use of particular services. For example, informal care can substitute for formal care for older people.

The purpose of our study was to identify patient-level characteristics and municipality-level service patterns that may be associated with the use and costs of health and social services for frail elderly people.

## Methods

### Design and study population

The individual-level data were obtained from a randomised controlled trial concerning a geriatric rehabilitation programme designed for frail elderly persons [21,22], which was conducted during 2002–2007 (Age-Study). The inclusion criteria were age of 65+ years, progressively decreasing functional ability, and risk of institutionalization within 2 years. The definition of frailty is based on the Pensioners’ Care Allowance benefit granted by the Finnish Social Insurance Institution (SII). This definition covers biological, physiological, social, and environmental changes. Our analysis is based on a sample of 732 frail elderly persons living in 41 municipalities. The sample characteristics are presented in Table 1.

**Table 1:** Characteristics of the study population

**Variable**	**Total Sample**	**Age<75 years**	**Age≥75 years**	**p-values**
	**n = 732**	**n = 215**	**n = 517**	
Age, mean (SD)	78 (6.4)	70 (2.8)	82 (4.7)	<.0001
Male, n (%)	101 (14)	61 (28)	40 (8)	<.0001
GDS, mean (SD)*	4.2 (2.5)	4.1 (2.5)	4.2 (2.5)	0.5191
Depressive mood				
GDS 7–13, n (%)	131 (18)	38 (18)	93 (18)	0.9196
MMSE, mean (SD)†	25 (2.9)	26 (2.8)	25 (2.9)	<.0001
Declined cognitive capacity				
MMSE <24, n (%)	210 (29)	44 (20)	166 (32)	0.0015
HRQOL 15D, mean (SD)‡	0.73 (0.1)	0.74 (0.1)	0.73 (0.1)	0.4588
FIM, mean (SD)§	116 (7.9)	116 (8.6)	115 (7.6)	0.2337
Self rated health, n (%)				0.8778
Very good	2 (0.3)	1 (0.5)	1 (0.2)	
Quite good	27 (4)	7 (3)	20 (4)	
Neither good nor poor	477 (65)	143 (67)	334 (65)	
Rather poor	209 (29)	58 (27)	151 (29)	
Very Poor	17 (2)	6 (3)	11 (2)	
Widowed, n (%)	455 (62)	81 (38)	374 (72)	<.0001
Living alone, n (%)	527 (72)	138 (64)	389 (75)	0.0024
Living in an urban area, n (%)	511 (70)	151 (70)	360 (70)	0.9006
Perceiving health deterioration during preceding year, n (%)	484 (66)	138 (64)	346 (67)	0.4758
Informal care, n (%)				0.1237
Yes	535 (73)	146 (68)	389 (75)	
No	91 (12)	32 (15)	59 (12)	
Missing information	106 (15)	37 (17)	69 (13)	
Formal home help				
visits/week, mean (SD)	1.9 (4.9)	2.0 (6.2)	1.8 (4.2)	0.7328
Hospital admissions, n (%)	365 (50)	101 (47)	264 (51)	0.2802
Visits to general				
practitioner, mean (SD)	4 (3.9)	4 (3.9)	4 (3.9)	0.9314

* GDS: Geriatric Depression Scale, max 15, values 0–6 indicate non-depression.

† MMSE: Mini Mental State Examination, max 30, values under 24 indicate existence of dementia.

‡ 15D: Health-related quality of life (HRQoL), range 0–1, 1 indicates the best imaginable health.

§ FIM: Functional Independence Measure, max 126, three subscales (Self Care 8 items, Mobility 5 items, Cognition 5 items) were formed from 18 items (range: 1 = total assistance – 7 = complete independence).

The present study was approved by the Ethical Committees of the SII and Turku University Hospital. All of the study participants gave their written consent to the study. The individual-level data were linked with data from the national databases of the Care Registers of the National Institute for Health and Welfare (THL) and the Social Insurance Institution (SII). The registry data aren’t publicly available.

### Utilization of services

The utilization of health care services covered both the public and private sector care. Data on inpatient care and day case surgeries were drawn from the National Care Registers of the THL. Data on the use of outpatient care services within the private sector and the use of medicines were obtained from the SII registers. A self-reported questionnaire was used to collect information about the number of visits to the public sector outpatient care during the preceding year.

The utilization of social services covered institutional care and formal home-help services provided by municipalities. For those living in residential homes and sheltered housing, services such as home help, washing and cleaning were included in the analysis. For those who were living at home, formal home care, home nursing, and support services were included. Data on the utilization of social care services were obtained from questionnaires. We asked the municipal social and health care officials to collect data on service use from their clients’ individual care and service plans. The data derived from questionnaires were cross-sectional both at baseline and 12-month follow-up. For those cases where changes occurred in the use of services during the follow-up, the annual data consisted 6 months of services used at the baseline and 6 months of services used at the follow-up.

The total expenditure of health and social services utilization was determined by multiplying the use of services by their standard unit costs. For the monetary valuation of health and social care services, we used Finnish standard costs information [23]. Because we used standard costs, any variation in the costs variables resulted from differences in the utilization of services.

To estimate the effects of independent characteristics on the costs of service utilization, we used the following three models:

Model 1: The costs of health care services and medicines (continuous variable, logarithm adjusted euro/year)

Model 2: The use of social care services (categorized into two groups; ‘yes’ if service use was observed and ‘no’ if otherwise)

Model 3: The costs of social care services used (categorized into three groups; under 1500, 1500–6000, or over 6000 euro/year)

Due to the fact that informal care is a common way to organize care for elderly, there were 240 subjects how did not use social services at all (‘No’ in MODEL 2). For those who used social services, living in sheltered housing causes stairwise effect to costs. Because the distribution was rising in steps and the assumptions of continuity did not hold, we categorized the costs of social care (MODEL 3).

### Functional assessments

Functional ability was assessed by the Functional Independence Measure (FIM), with the total score ranging from 18 (the lowest) to 126 (the highest level of independence) [24], and by the subject’s ability to perform the instrumental activities of daily living as measured by the IADL index, which varies between 7 and 21 (the higher the score, the more difficulties). Mood was measured by the Geriatric Depression Scale (GDS), where the maximum value is 15 and values 0–6 indicate non-depressiveness [25]. Cognitive capacity was measured by the Mini Mental State Examination (MMSE), with the maximum value being 30 and values under 24 indicating dementia [26]. The assessments were carried out by three accredited examiners.

Health-related quality of life (HRQoL) was evaluated by using the 15D score, ranging 0–1 (‘1’ indicates the best imaginable health) [27,28]. To estimate the impact of recent changes in health status, the subjects were asked “How has your health status changed during the last year?” Three class variables were created from the five alternative responses: worse (“much worse”, “worse”), same (“no change”), and better (“better”, “much better”). The questionnaires were checked by the examiner and any incomplete sections were completed by interviewing the participants.

### Living conditions

To assess the living conditions, the subjects were asked “Is your residential environment rural or urban?” (yes/no) and “Are you living alone?” (yes/no). The dwelling circumstances were categorized into three categories: home without formal home-help, home with formal home-help, and residential home or sheltered housing.

### Informal help

The municipal social and health care officials were asked to collect information on informal support and assistance from their clients’ personal care and service plans. They reported "yes" if the person received informal care that was considered as supplemental to formal care, and "no" if otherwise.

A self-reported questionnaire was presented to the subjects to collect information on the availability and extent of informal care. Respondents were asked to answer the question “Is there any person who helps you when needed?” The answers were categorized into four categories; not available, occasionally, when needed shortly, as long as needed. Respondents were also asked whether they had been granted a Home Care Allowance by their local municipality.

### Rehabilitation

For the purposes of the Age Study, the subjects were randomly assigned to either an in-patient rehabilitation programme or standard social and health care. In this study, to standardize the effect of the rehabilitation programme, we included rehabilitation as an explanatory variable in all our models.

### Administrative integration of the health and social care sectors at municipal level

At municipal level, we investigated if municipalities had integrated the administration of their health care and social services (joint budgets and management). We asked the municipal social and health care officials: “Is your local health and social care administration integrated or not?” (yes/no).

### Statistical analyses

A hierarchical analysis with fixed (patient level) and random (municipality level) effects was used to estimate the effects of explanatory variables on the utilization and costs of health and social services. We used the SAS PROC GLIMMIX procedure to fit the models [29,30].

First, bivariate correlations between independent variables were examined to check for correlations. All correlations were low. The independent variables were included in the multivariate model using the entrance value of P < 0.05 in the univariate analysis. All multivariate analyses were adjusted for rehabilitation in order to standardize the effect of the rehabilitation in the randomized trial setting. The effect sizes as a result of linear and logistic analyses were expressed as estimates (β) or odds ratios (OR) or cumulative odds ratios (COR) with 95% confidence intervals (CL) and the corresponding p-values.

For quantitative variables, skewness of distribution was analysed. Skewness was detected in health care costs, and a logarithm transformation was calculated to correct the distribution. In the analysis, we divided the variables concerning functional needs (FIM, GDS, MMSE, 15D, IADL) by their standard deviation to make them commensurable and to make it easier to compare the odds ratios. Age was divided by five in order to highlight the impact of every five-year increment in age.

## Results

### Baseline characteristics of the subjects

At the baseline, the mean age of the subjects was 78 years (range 65–96 years). In the older age group (over 75-year-old subjects), the number of people living alone was higher and impaired cognitive capacity was found more often. Informal care and hospital admissions were also more common in the over 75-year-olds (Table 1). Due to these differences in need-specific factors, the over 75-year-olds were the largest client group for home care services.

### Costs of the utilization of health and social care

The mean total costs of the utilization of health and social services were 10300 euro during one year (median 5400 euro) (Table 2). The proportion of health care costs was, on average, 48% of the total costs. Altogether 492 subjects had used social care services during the preceding year, and the mean costs of their social service utilization were 8700 euro (median 4200 euro) per year.

**Table 2:** Costs of the utilization of health and social services (Euro)

	**N**	**Mean**	**Median**	**(Q25%, Q75%)**
Total costs	732	10300	5400	(2200, 12300)
Health care costs	731	4500	2400	(1100, 5300)
Social care costs	492	8700	4200	(1200, 10600)

### Variance components in intercept-only model

An analysis was carried out in two steps. First, an intercept-only model without any explanatory variables was applied. The intraclass correlation of the total costs of health and social care services was 0.06, meaning that roughly 6% of the variance is attributable to the municipality level and 94% to the patient level. When costs were examined for the health care and social care sectors separately, the variance in the costs of health care services was totally explained by individual differences in health; the covariance estimate for the municipality variable was zero. In the use of social services, a null model with two levels showed that 9% of the variability in the social services costs was explained by the municipality level and 91% by the patient level.

### Factors associated with the costs of health care services utilization (Model 1)

In the multivariate model, the health-related quality of life (15D score) appeared to be a powerful indicator for utilization of health care services (p = 0.0004) (Table 3). A statistically significant decline in HRQol 15D (0.02 units) leads to 1.5 folds increase in costs. The association between other explanatory factors and health care utilization was examined by fitting the multivariate model without 15D score. Independent living and better functional ability (FIM) decreased health care utilization (not tabulated).

**Table 3:** Results of regression, logistic or cumulative logistic analysis showing the regression coefficients (β), odds ratios (OR) and cumulative odd ratios (COR) with 95% confidence intervals (CI) for health and social care costs and social care utilization

	**Model 1**				**Model 2**				**Model 3**			
	**Logarithm adjusted health care costs**				**Social care utilization**				**Cost of social care**			
	Continuous variable				Categorized into groups; yes, no				Categorized into groups; under 1500, 1500-6000 or over 6000 €/year			
	n = 732				n = 732				n = 492			
	**Univariate**		**Multivariate**		**Univariate**		**Multivariate**		**Univariate**		**Multivariate**	
	**β**	**(Cl 95%)**	**β**	**(Cl 95%)**	**OR**	**(Cl 95%)**	**OR**	**(Cl 95%)**	**COR**	**(Cl 95%)**	**COR**	**(Cl 95%)**
**Patient level**												
Rehabilitation												
*Intervention*	0.09	(-0.08, 0.26)	0.09	(-0.08, 0.26)	0.86	(0.62, 1.18)	0.88	(0.62, 1.26)	1.09	(0.78, 1.52)	1.13	(0.78, 1.64)
*Control*	0		0		1		1		1		1	
Gender												
*Male*	-0.11	(-0.36, 0.14)			0.86	(0.54, 1.36)			1.70	(1.02, 2.82)*	1.43	(0.81, 2.55)
*Female*	0				1				1		1	
Age (five years)	0.02	(-0.04, 0.09)			1.41	(1.23, 1.60)*	1.30	(1.12, 1.51)*	1.12	(0.98, 1.28)		
Location of residence												
*Urban*	0.20	(0.02, 0.39)*	0.08	(-0.11, 0.28)	2.02	(1.4, 2.91)*	1.45	(0.96, 2.19)	1.92	(1.29, 2.88)*	1.19	(0.75, 1.88)
*Rural*	0		0		1		1		1		1	
Living conditions †												
*Home*	-0.34	(-0.70, 0.01)*	-0.15	(-0.51, 0.22)					0.03	(0.01, 0.06)*	0.06	(0.03, 0.14)*
*Home with home help*	-0.05	(-0.40, 0.30)	0.00	(-0.34, 0.35)					0.37	(0.19, 0.70)	0.48	(0.25, 0.95)
*Sheltered housing or residential home*	0		0						1		1	
Living alone												
*Not alone*	-0.26	(0.45, -0.07)*	-0.14	(-0.36, 0.07)	0.33	(0.23, 0.48)*	0.41	(0.27, 0.62)*	0.29	(0.19, 0.45)*	0.48	(0.28, 0.80)*
*Alone*	0		0		1		1		1		1	
Availability of informal caregiver												
*Not available*	0.18	(-0.11, 0.46)			1.36	(0.80, 2.29)*	1.24	(0.68, 2.27)*	1.58	(0.89, 2.81)		
*Occasionally*	0.26	(0.02, 0.50)			2.02	(1.26, 3.22)	2.02	(1.17, 3.46)	1.45	(0.91, 2.31)		
*When needed shortly*	0.14	(-0.07, 0.35)			1.95	(1.30, 2.91)	1.68	(1.06, 2.65)	1.46	(0.96, 2.21)		
*As long as needed*					1		1		1			
Receiving informal help												
*No*	0.15	(-0.05, 0.36)			1.33	(0.89, 1.98)			2.03	(1.35, 3.06)*	1.79	(1.12, 2.86)*
*Yes*	0				1				1		1	
Home care allowance												
*No*	0.09	(-0.33, 0.51)			0.99	(0.45, 2.19)			3.62	(1.54, 8.50)*	2.80	(1.01, 7.76)*
*Yes*	0				1				1		1	
Self-assessed change in health during preceding year												
*Better*	-0.25	(-0.59, 0.08)*	-0.16	(-0.50, 0.18)	2.82	(1.27, 6.24)*	3.81	(1.54, 9.40)*	1.07	(0.60, 1.93)		
*Same*	-0.30	(-0.50, -0.01)	-0.13	(-0.34, 0.08)	0.93	(0.65, 1.34)	1.22	(0.79, 1.90)	1.32	(0.89, 1.95)		
*Worse*	0		0		1		1		1			
IADL(SD)^1^	0.11	(0.02, 0.19)*	-0.01	(-0.04, 0.02)	1.44	(1.21, 1.72)*	1.29	(1.02, 1.64)*	1.92	(1.59, 2.31)*	1.60	(1.24, 2.06)*
GDS(SD)^2^	0.13	(0.04, 0.22)*	0.00	(-0.04, 0.04)	1.46	(1.22, 1.74)*	1.28	(1.04, 1.58)*	1.27	(1.06, 1.51)*	1.15	(0.93, 1.42)
MMSE(SD)^3^	0.03	(-0.06, 0.12)			0.74	(0.62, 0.88)*	0.81	(0.67, 0.98)*	0.82	(0.70, 0.97)*	0.90	(0.75, 1.08)
15D(SD)^4^	-0.26	(-0.34, -0.17)*	-1.90	(-2.94, 0.85)*	0.72	(0.61, 0.86)*	1.00	(0.78, 1.27)	0.84	(0.71, 1.00)*	1.19	(0.96, 1.48)
FIM(SD)^5^	-0.18	(-0.27, -0.09)*	-0.01	(-0.03, 0.00)	0.51	(0.41, 0.63)*	0.63	(0.48, 0.82)*	0.54	(0.44, 0.65)*	0.77	(0.61, 0.98)*
**Municipality level**												
Combined health and social sectors in municipal												
*Yes*	0.07	(-0.10, 0.25)			1.09	(0.68, 1.76)			0.65	(0.42, 1.00)		
*No*	0				1				1			

*= P<0.05.

^†^ Living conditions is not included in the models of social care utilization because it is logically too close to the despondence variable.

^1^ Coefficient corresponding change of standard error. The higher IADL scores indicates more difficulties.

^2^ Coefficient corresponding change of standard error. The higher GDS scores indicates more more depressiveness.

^3^ Coefficient corresponding change of standard error. The higher MMSE scores indicates less demented.

^4^ Coefficient corresponding change of standard error. The higher 15D scores indicates better health.

^5^ Coefficient corresponding change of standard error. The higher FIM scores indicates highest level of independence.

Costs of the utilization of health and social services.

### Factors associated with social care services utilization (Model 2)

A logistic analysis was conducted to explain the use of social care services (Table 3). In the multivariate analysis, improvement in health status during the preceding year was associated with social services utilization. Instrumental activities of daily living (IADL), mood (GDS), dementia (MMSE) and functional ability (FIM) had a major impact on the use of social care services. Location of residence and health-related quality of life were not significantly related to the presence of social services utilization in the multivariate model. Every five-year increment in age and living alone increased the probability of social services utilization. Availability of informal care reduced the probability.

### Factors associated with the costs of social care services utilization (Model 3)

The costs of received social care services were categorized into three groups: annual costs under 1500, 1500–6000, and over 6000 euro (Table 3). When controlled for the confounding effect of the explanatory variables with a multivariate model, the caregiver’s financial support was most strongly associated with lower costs of social service use. Receiving informal care was also associated with a lower amount of used services. Poor ability in instrumental activities of daily living and low level of functional performance were associated with higher costs of service use. Living in a residential home or sheltered housing and living alone were associated with higher costs. The entrance value to multivariate analysis was P < 0.05, but we conducted the analysis also with the integrated social and health care sector variable included. The integrated social and health care sector was not significantly related to costs in the multivariate setting (not tabulated).

## Discussion

We found that, problems of access to services were concentrated in social care services rather than in health care in frail elderly population in Finland. No differences were observed in the utilization of health care services at municipality level, whereas in the social services, 9% of the variation was explained by the municipality-level factors. Since informal care could be substituting for formal social care services, it is possible that differences in the municipalities’ policies to support informal care explain, to some extent, the variability in social service use.

At the patient level, we found that living alone and availability of informal care were strong explanatory variables of the costs of social services. There is a growing trend that older people live alone, and the female informal care-giving potential is declining due to rising female employment rates [31]. A reduction in informal care can have a major impact on the demand for formal care in the future. Informal care and single households are likely to be important determinants of future long-term care expenditure.

In our study, a connection was found between the utilization of social care services and perceived health. Those who reported improvement in their health status during the preceding year were more frequent users of social care services. According to Andersen, effective access to care is established when use of services is connected with improves in health status [5]. Our finding supports the evidence that if a person receives adequate support and social care services, his or her need for health care services may be reduced [32]. Currently, the municipalities are cutting down support services and targeting services only for those who are in the highest need, which may, in the long run, result in increased costs of health care. Earlier studies have shown that ADL-dependent subjects and those at risk of depression had significantly more hospital stays [19].

This is consistent with the literature emphasizing the significance of integrated care for the elderly [33-35]. Still, when need factors were controlled for, municipalities with integrated health and social care sectors had similar patterns in access to care and similar costs as compared to municipalities without such integration.

Our findings indicate that the administrative structure alone does not ensure that integration of care has positive effects. Earlier studies have suggested that community based integrated health and social care services might better meet the multiple demands of the population, especially among frail elderly population [33,36,37]. Based on our results, it seems that success of integration may depend on the combination of methods and strategies selected to achieve this objective.

### Methodological considerations

In the multivariate models, we introduced explanatory variables mostly at the patient level. At the municipality level, we focused on the question of integrated services. We measured integration with one question concerning the administrative integration, and this may be insufficient to describe the multidimensional phenomena of integration.

No other specific variables – such as proportion of elderly people, economical situation, or population size – were introduced at the municipality level, because we did not aim to explain differences between municipalities. Such variables should, however, be taken into consideration in future studies.

While frail aged people with deteriorated health must be provided care as soon as any need occurs, the results of this study cannot necessarily be generalized to apply to the aged population as a whole.

In lack of national registers, data on municipal primary health care and social care services were collected through questionnaires that were completed by the elderly participants and by the representatives of the local social and health care units. In Finland, municipalities are the main provider of services for the elderly, and therefore, municipal records on service use are comprehensive and reliable. Furthermore, a vast majority of the costs were calculated from registry data, which in Finland are regarded to be very reliable [38].

## Conclusion

Municipality-level variation was observed in the utilization of social services, whereas health care services provided for frail elderly people seem to be highly equitable at the municipal level. Nevertheless, there may be differences in access to services at the patient level due to factors which we did not measure, for example, socio-economic factors.

We found that the utilization of social and health services were connected. Those who reported improvement in their health status during the preceding year were more frequently also using social services. This result suggests that if municipalities continue to limit the provision of support services only for those who are in the highest need, this saving in the social sector may, in the long run, result in increased costs of health care. In order to control the expenditure, it is necessary to ensure that the integration of services provided for the elderly residents is not only an administrative but also a practical measure. Another important determinant of care expenditure is informal care. Informal care and single households can have a major impact on the demand for formal care in the future.

## Competing interests

The authors have no financial or non-financial competing interests.

## Authors' contributions

SK participated in the design and coordination of the study, performed the statistical analysis and drafted the manuscript. IA-R participated in the design and coordination of the study and drafted the manuscript. KH participated in the design and coordination of the study and helped draft the manuscript. HH tutored and participated in the statistical analysis and helped draft the manuscript. MV participated in statistical analysis and helped draft the manuscript. PR participated in the design of the study and drafted the manuscript. All authors have read and approved the final manuscript.

## Pre-publication history

The pre-publication history for this paper can be accessed here:

http://www.biomedcentral.com/1472-6963/12/204/prepub
